# Right-sided Bochdalek hernia in an adult: a case report

**DOI:** 10.1093/jscr/rjab357

**Published:** 2021-08-28

**Authors:** Ashis Pun, Pratibha Dhoubhadel, Kamal Dawadi

**Affiliations:** MS General Surgery, Department of General Surgery, Bharatpur Hospital, Chitwan, Nepal; MS General Surgery, Department of General Surgery, Bharatpur Hospital, Chitwan, Nepal; MD Internal Medicine, Department of Internal Medicine, Hetauda Hospital, Makwanpur, Nepal

## Abstract

Bochdalek hernia (BH) is the most common type of congenital diaphragmatic hernia and is usually left-sided. It typically presents in neonates and diagnosis in adults is a rarity. To date only 34 reported cases of right-sided BH have been surgically managed in adults in literature. We review a 21-year-old female with a right-sided BH diagnosed as acute calculus cholecystitis and underwent laparoscopic cholecystectomy. The diagnosis of BH was made incidentally during surgery. The hernia did not have a sac with no hernial contents. It was treated via laparoscopic intracorporeal suturing and patient postoperative period was uneventful. Right-sided BH is rare. Regardless of the symptoms surgical treatment should be done to avoid risk of visceral strangulation.

## INTRODUCTION

First described in 1848 by Vincent Alexander Bochdalek, Bochdalek hernia (BH) is a congenital posterior diaphragmatic defect due to failure of closure of the posterolateral aspect of the pleuroperitoneal canal with septum transversum at around 6 weeks of gestation [1].

Presenting commonly in childhood the prevalence is 1:2000 live births and present in <0.2% of adults. Childhood presentation includes respiratory failure, pulmonary hypertension with a risk of congenital developmental anomalies in 10–15% [2]. Due to the rapid advances in imaging modality BH is being more commonly reported [3]. In adults a precipitating factor such as obesity, exertion, trauma and pregnancy may be present. Clinical manifestations such as pain, strangulation, obstruction, pulmonary symptoms, bleeding and dysphagia may occur, pain being the most common. Only ~14% of patients are asymptomatic at the time of presentation [4].

To date only 34 reported cases of right-sided BH have been surgically managed in adults [5]. We report one additional case of right-sided BH.

## CASE REPORT

A 21-year-old female presented with pain in the right upper abdomen for 1 day in the emergency department. She had no history of any thoracic or abdominal trauma and no comorbidities. She was evaluated and treated for pain. Investigations revealed normal blood count and liver function tests. Ultrasonography of abdomen and pelvis revealed distended gall bladder with gall stone. Chest X-ray revealed elevated right hemi diaphragm. She was diagnosed as acute calculus cholecystitis and planned for laparoscopic cholecystectomy.

**Figure 1:**
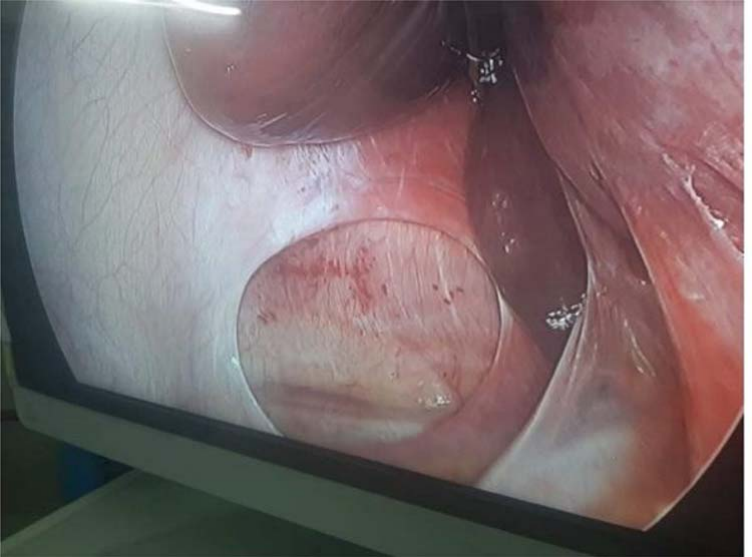
Laparoscopic image of right BH with no sac.

**Figure 2:**
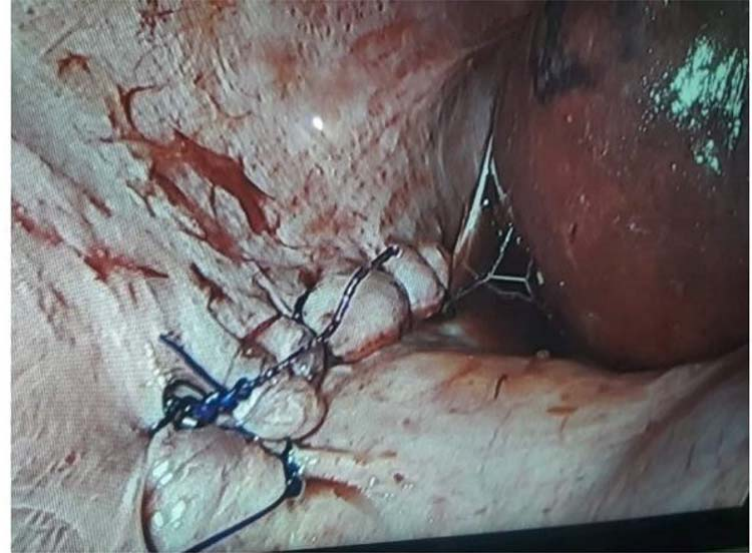
Image after repair of hernia defect.

Laparoscopic cholecystectomy was performed under 12 cm H_2_O pressure of pneumoperitoneum. Intraoperatively, a ~4-cm defect was noted in the right hemidiaphragm. The hernial defect was devoid of hernial sac and had no contents. An intraoperative diagnosis of right-sided BH was made. After laparoscopic cholecystectomy was performed we repaired the hernial defect using a single layer intracorporeal suturing using V-loc sutures, however no mesh was used in the repair process because of infective gallbladder pathology (Figs 1 and 2).

No intra- or postoperative pneumothorax was noted. The postoperative course was uneventful and the patient was discharged 2 days postoperatively. On subsequent follow-up at 2 months, the patient reported good quality of life and had no symptoms or recurrence.

## DISCUSSION

BH are rare in the right side compared with the left-side (80–90%) owing to the earlier closure of the right canal and protective presence of the liver [6].

In a study done by Brown *et al.*, 124 articles comprising of 173 patients were reviewed in which 78% patients had left-sided defect, 20% right side and 2% had bilateral BH. In contrary, Mullins reported a review of 13 138 abdominal CT reports that the incidence of adult BH was 0.17%, with 68% being right-sided and 77% of patients being female. This suggests the silent nature of right-sided BH. Right sided BH are also under reported due to lack of clinical symptoms [7].

Also some form of inciting event such as pregnancy, exertion, retching and trauma was noted in 25% of the cases presenting with symptomatic BH [4] however our patient did not have any inciting event.

Regarding radiological evaluation the only suggestive finding was a slightly elevated right hemidiaphragm. However a normal X-ray film of the chest should not rule out BH entirely. Various other investigations like computed tomography (CT) scan and magnetic resonance imaging (MRI) can more accurately diagnose BH. A high degree of clinical suspicion along with supportive investigation can correctly diagnose BH.

In our patient hernia sac was absent. In a study done by Brown *et al.* out of 173 patients only 18 had hernial sac. Literature shows that hernial sac is more common in Morgagni hernia and is rare in BH [4]. If a hernia sac is present excision of the sac and repair of defect with interrupted non-absorbable sutures is recommended. Whenever a hernia sac is absent the free communication between pleural and peritoneal spaces increases risk of postoperative pneumothorax when laparoscopy is used [3]. However no such complication was noted in our patient.

Various surgical repair options include open surgery, laparoscopic repair, thoracoscopic approach and robotic transthoracic approaches. Best approach is yet to be determined with further large scale studies. Laparoscopy is minimally invasive and provides better visualization and working space than an open approach especially when the right hepatic lobe obstructs view in right-sided BH [8]. Some studies state that there is better visualization and working space for separation of adhesions between herniated contents and thoracoscopic structures during thoracoscopic approach [9]. Others advocate that the refined optics of robotic system have an added advantage [10]. Most authors state that regardless of the symptoms of BH, surgical repair with or without use of prosthetic mesh or muscle flap should be done prophylactically to avoid the risk of visceral incarceration. Our patient was managed laparoscopically using V-Loc suture.

In conclusion, right-sided BH is a very rare condition, however its incidental detection is increasing due to the rapid advances in imaging. BH should be managed timely regardless of its symptoms to avoid future complications. It can be successfully managed using laparoscopic techniques, further studies need to be done to determine the best approach.
