# Right-sided Bochdalek hernia in an adult: a case report

**DOI:** 10.1093/jscr/rjab432

**Published:** 2021-09-23

**Authors:** Ashis Pun, Pratibha Dhoubhadel, Kamal Dawadi

**Affiliations:** 1 MS General Surgery, Department of General Surgery, Bharatpur Hospital, Chitwan, Nepal; 2 MD Internal Medicine, Department of Internal Medicine, Hetauda Hospital, Makwanpur, Nepal

When this paper first published, an author name was incorrectly spelled as “Asish Pun”. This should have been “Ashis Pun”. This error has now been corrected online.

